# Protective effects of aucubin on osteoarthritic chondrocyte model induced by hydrogen peroxide and mechanical stimulus

**DOI:** 10.1186/s12906-017-1581-y

**Published:** 2017-02-02

**Authors:** In-Chi Young, Sung-Ting Chuang, Chia-Hsien Hsu, Yu-Jun Sun, Hwa-Chang Liu, Yo-Shen Chen, Feng-Huei Lin

**Affiliations:** 1 Institute of Biomedical Engineering, National Taiwan University, No. 49, Fanglan Rd, Taipei, 10672 Taiwan; 2 Institute of Pharmacology, College of Medicine, National Taiwan University, No. 1, Sec. 1, Ren-Ai Rd, Taipei, 10051 Taiwan; 3 Institute of Biomedical Engineering and Nanomedicine, National Health Research Institute, No. 35, Keyan Rd, Miaoli, 35053 Taiwan; 4 Department of Orthopaedic Surgery, Taiwan Adventist Hospital, No. 424, Sec. 2, Bade Rd, Taipei, 10556 Taiwan; 5 Department of Orthopaedic Surgery, National Taiwan University Hospital, No.7, Chung Shan S. Rd, Taipei, 10002 Taiwan

**Keywords:** Aucubin, Osteoarthritis, ROS, Mechanical stress, Inflammation

## Abstract

**Background:**

During the onset of osteoarthritis (OA), certain biochemical events have been shown to accelerate cartilage degradation, including the dysregulation of cartilage ECM anabolism, abnormal generation of reactive oxygen species (ROS) and overproduction of proteolytic enzymes and inflammatory cytokines. The potency of aucubin in protecting cellular components against oxidative stress, inflammation and apoptosis effects are well documented, which makes it a potential candidate for OA treatment. In this study, we aimed to evaluate the protective benefits of aucubin against OA using H_2_O_2_ and compression induced OA-like chondrocyte models.

**Methods:**

The effects of aucubin were studied in porcine chondrocytes after 1 mM H_2_O_2_ stimulation for 30 min or sustained compression for 24 h. Effects of aucubin on cell proliferation and cytotoxicity of chondrocytes were measured with WST-1 and LDH assays. ROS production was evaluated by the Total ROS/Superoxide Detection Kit. Caspase-3 activity was evaluated by the CaspACE assay system. The levels of apoptosis were evaluated by the Annexin V-FITC apoptosis detection kit. OA-related gene expression was measured by reverse transcription quantitative polymerase chain reaction (RT-qPCR). Total DNA quantification was evaluated by the DNeasy Blood and Tissue kit. Sulfated-glycosaminoglycans (sGAGs) production and content were evaluated by DMMB assay and Alcian blue staining.

**Results:**

The results showed that the ROS scavenge effects of aucubin appeared after 1 h of pretreatment. Aucubin could reduce the caspase-3 activity induced by H_2_O_2_, and reduced the apoptosis cell population in flowcytometry. In RT-qPCR results, aucubin could maintain ACAN and COL2A1 gene expressions, and prevent IL6 and MMP13 gene up-regulation induced by H_2_O_2_ and compression stimulations. In the DMMB assay and Alcian blue staining, aucubin could maintain the sGAG content and protect chondrocytes against compressive stress, but not oxidative stress from H_2_O_2_.

**Conclusions:**

These results indicated that aucubin has protective effects in an osteoarthritic chondrocyte model induced by H_2_O_2_ and mechanical stimulus.

## Background

Osteoarthritis (OA) is the most common form of arthritis and the leading cause of disability in people over 65 years old [1, 2]. The high prevalence rate among the elderly makes it a considerable clinical and economic burden because of reduced quality of life and increased use of health care resources [3, 4]. OA involves the entire joint, including the subchondral bone, ligaments, periarticular muscle and synovium, and is associated with risk factors, such as age, gender, prior joint injury, obesity, genetic predisposition and mechanical stress [5, 6]. In healthy individuals, synthesis and degradation of cartilage extracellular matrix (ECM) maintains a particular balance. However, this homeostatic balance can be disrupted in OA cartilage by reduced anabolic and increased catabolic capacities of chondrocytes. Chondrocytes are the unique cells of the articular cartilage ECM and are responsible for the synthesis and degradation of the cartilage, which mainly consists of type II collagen and sulfated proteoglycans (sGAG) [7]. During the initiation of OA, biochemical events have been shown to accelerate the cartilage degradation, including the dysregulation of the ECM anabolism, abnormal generation of reactive oxygen species (ROS) and overproduction of proteolytic enzymes and inflammatory cytokines [8–10]. Disruption of homeostasis decreases type II collagen and sGAG and leads to the loss of cellularity via apoptosis, which plays a central role in the caspase proteolytic cascade, all of which is evident in the OA cartilage [11].

Damage from mechanical stress with insufficient self-repair by joints is believed to be the primary cause of OA. Excessive compressive stress can increase the production of ROS in chondrocytes, which is sufficient to depolymerize hyaluronic acid of ECM [12] or even kill chondrocytes [13]. In response to mechanical loading of articular cartilage, chondrocytes exhibit the same changes in gene expression as those during OA, such as upregulation of the matrix metalloproteinase 13 gene (MMP13) and a disintegrin and metalloproteinase with thrombospondin motifs (ADAMTS) gene family. Therefore, cartilage explants stimulated with mechanical stresses have been used as typical experimental models [14].

Several studies have shown that ROS, such as superoxide anions, hydroxyl radicals and hydrogen peroxide (H_2_O_2_), play a role in cartilage degeneration [15, 16]. A high-level of ROS production commonly results in apoptosis and senescence of the chondrocytes, and thus associates with a decreased number of chondrocytes and altered cell phenotypes during OA [17]. H_2_O_2_ is a potent mediator of membrane lipid peroxidation. The disruption of mitochondrial membrane integrity caused by H_2_O_2_ leads to the release of caspases that play essential roles in apoptosis [18]. Thus, H_2_O_2_ is frequently used as an inducer of oxidative stress to investigate the role of cellular antioxidants, like vitamin E, in ameliorating cellular injury [19].

In our previous studies, we have found that antioxidants from Chinese herbal medicines have potential benefits in long-term treatments of ROS-mediated diseases [20]. Aucubin, an iridoid glucoside isolated from various plants including leaves of *Aucuba japonica* and *Eucommia ulmoides* [21, 22], has proven to possess numerous pharmacological effects [23–25]. The potential role of aucubin in protecting cellular components against oxidative stress and inflammatory responses is well documented [26, 27]. However, the effect of aucubin on the catabolic responses of chondrocytes or its therapeutic role in OA has not been identified.

In this study, we investigated the effects of aucubin on the following gene expressions: ECM-related genes (COL2A1 and ACAN), the catabolic MMP13 gene and the proinflammatory cytokine IL6 gene. The effect of aucubin on ROS production, caspase-3 activity, chondrocyte apoptosis, cell proliferation activity and cytotoxicity, sGAG production and sGAG content were also investigated. Finally, we discuss the potential utility of aucubin as a treatment for OA in light of our findings.

## Methods

### Isolation of chondrocytes

The isolation and use of porcine chondrocytes were approved by the Animal Experimentation Ethics Committee of National Taiwan University Hospital. Fresh porcine stifles were purchased from a traditional market and kept integrated till chondrocytes were isolated under aseptic conditions. A total of 12 porcine stifles were used for all experiments. Porcine chondrocytes were isolated from macroscopically normal cartilage of the femoral condyles [28]. Finely diced cartilage pieces were treated with 10% antibiotics (15240-062, Gibco, USA) in phosphate buffered saline (PBS) at 37 °C for 10 min, then re-suspended in Dulbecco’s modified eagle’s medium (DMEM; D5648, Sigma, USA) containing 10% fetal bovine serum (12003C, SAFC, USA), 1% penicillin and 0.05% L-Ascorbic acid (A5960, Sigma, USA) and 0.2% collagenase (C0130, Sigma, USA) at 37 °C for 18 h. Chondrocytes were then collected and washed twice with PBS, and cultured in DMEM. Chondrocytes with a passage number of 2 to 4 were used in all experiments.

### Cell proliferation and cytotoxicity of aucubin on chondrocytes

Chondrocytes were seeded in 96-well cell culture plates at a density of 1 × 10^4^ cells per well and cultured in DMEM for 18 h. Cells were then cultured in the medium containing 3, 10, 30, 100 μM aucubin (55561, Sigma, USA). To evaluate the cell proliferation activity of aucubin on chondrocytes, WST-1 assay (Cell Proliferation Reagent WST-1; Roche, Germany) was performed on days 1 and 3. The OD value was measured at 450 nm using the enzyme-linked immunosorbent assay (ELISA) reader (Sunrise, Tecan, Switzerland).

The LDH assay (CytoTox96 Non-Radioactive Cytotoxicity Assay; Promega, USA) was performed on days 1 and 3 to evaluate the cytotoxicity of aucubin. LDH released in the culture supernatants was measured with a 30-min coupled enzymatic assay and measured using the ELISA reader at a wavelength of 490 nm. The percentage of cytotoxicity was calculated using the following equation:

$$ \mathrm{Cytotoxicity}\ \left(\%\right) = \frac{{\mathrm{OD}}_{\exp } - {\mathrm{OD}}_{\mathrm{medium}}}{{{\mathrm{OD}}_{\mathrm{total}}}_{\mathrm{lysis}} - {\mathrm{OD}}_{\mathrm{medium}}} \times 100 $$

For both WST-1 and LDH assays, Chondrocytes derived from more than three porcine stifles were used for the experiments. Experiments were performed in five repeated measurements.

### Aucubin pretreatment and induction of oxidative stress

The chondrocytes were seeded in 6-well cell culture plates with a density of 1 × 10^5^ cells per well, cultured in DMEM with 10% fetal bovine serum (Gibco, USA) and incubated overnight. Cells were pretreated with 100 μM aucubin in full medium for 24 h. After washing once with PBS, the oxidative stress was induced by introducing 1 mM H_2_O_2_ (RDH, USA) in full medium for 30 min, which was followed by a second PBS wash, and then cultured in fresh medium at 37 °C for 24 h.

### ROS scavenge effect

ROS production was evaluated by Total ROS/Superoxide Detection Kit (ENZ-51010, Enzo Life Sciences, USA). After H_2_O_2_ treatment, chondrocytes were collected, washed twice with PBS and stained with 500 μl of the ROS detection mix for 30 min in the dark, and then analyzed with an ELISA reader (Spectra Max, Molecular Devices, USA). ROS fluorescence was also examined using a confocal laser scanning biological microscope (IX71/FV300, Olympus, Japan). Chondrocytes derived from more than three porcine stifles were used for the experiments. Experiments were performed in five repeated measurements.

### Caspase-3 activity

Chondrocytes were collected after H_2_O_2_ treatment. Total protein content was determined using a BCA protein assay kit (Pierce, USA) according to the manufacturer’s instructions. Caspase-3 activity was evaluated by a CaspACE assay system (Promega, USA). Thirty microgram of protein from each sample was mixed with a reaction buffer containing 2 μl of DMSO, 10 μl of 100 mM DTT and 32 μl of caspase assay buffer in a 96-well microplate. Two microlitre of DEVD-pNA was then added and the proteins in solution were incubated at 37 °C for 4 h. The absorbance was measured at the wavelength of 405 nm using an ELISA reader (Sunrise, Tecan, Switzerland). Chondrocytes derived from more than three porcine stifles were used for the experiments. Five repeated measurements were performed for all samples.

### Chondrocyte apoptosis

The chondrocyte apoptosis was evaluated by Annexin V-FITC apoptosis detection kit (ab14085, Abcam, USA). At the end of aucubin pretreatment and H_2_O_2_ stimulation, chondrocytes were collected, washed twice with cold PBS and stained with 500 μl of the Annexin V-FITC and PI mixed solution or 30 min in the dark, then analyzed by flow cytometry (FC500, Beckman, USA). Chondrocytes derived from more than three porcine stifles were used for the experiments. Five repeated measurements were performed for all samples.

### Induction of compressive stress

The compressive stress was introduced with a custom-made compressive device. We incorporated chondrocytes with chitosan-gelatin-glycerol phosphate hydrogel as cell carrier [29]. The hydrogel incorporated chondrocytes were seeded into the wells of the compression device (200 μl/well) and cultured at 37 °C. After 24 h of incubation, wells with cells were covered with polydimethylsiloxane membrane. The compression was given via nitrogen gas with pressure of 60 psi, which compressed the cells through the structural depression of membrane, and chondrocytes were subjected to sustained compression for 24 h. Chondrocytes without treatment were used as control group.

### RNA extraction and gene expression of chondrocytes

The chondrocytes were collected and total RNA was extracted using RNeasy Protect Mini kit (74104, QIAGEN, Germany). Total RNA yield and RNA quality were detected by spectrophotometer (NanoDrop™ 2000, Thermo Fisher Scientific, USA). RNA samples showed an A260/280 ratio between 1.8~2.0 and an A260/230 ratio between 2.0~2.2 were used for reverse transcription quantitative polymerase chain reaction (RT-qPCR). The first strand complementary DNA (cDNA) was synthesized from RNA and SuperScript™ III First-Strand Synthesis System (18080-051 Invitrogen, USA) according to the instructions provided by the manufacturer. The volume of the PCR Mix of single reaction was 20 μl containing 1 μl of primer solution, 9 μl of cDNA and 10 μl of 2× TaqMan Universal PCR Master Mix (4304437, ABI, USA). TaqMan Gene Expression Assays (Life Technology, USA) were used for gene expression analysis. The target genes of RT-qPCR are summarized in Table 1. RT-qPCR was performed using an ABI PRISM 7900HT Sequence Detection System and Sequence Detection Software 2.2.2. The target genes were normalized to the glyceraldehyde-3-phosphate dehydrogenase (GAPDH). The relative mRNA expression of each target gene was determined using the ^∆∆^Ct method.

**Table 1:** Primers used in this study

Target gene	Assay ID	GeneBank accession number
GAPDH	Ss03375435_u1	AF141959.1
COL2A1	Ss03373344_g1	AF201724.1
ACAN	Ss03374824_g1	X60107.1
MMP13	Ss03373279_m1	AF069643.1
IL6	Ss03384604_u1	AB194100.1

### Total DNA quantification

After compression/H_2_O_2_ treatment, chondrocytes were collected and total DNA was purified using the DNeasy Blood and Tissue kit (69504, QIAGEN, Germany), following the instructions provided by the manufacturer. Total DNA yield was quantified by an ultra violet/visible/near infrared (UV/VIS/NIR) spectrophotometer (DU 7500, Beckman, USA) at the wavelength of 260 and 280 nm. The ratio of 260 to 280 nm was between 1.8 and 2.0.

### Analysis of sGAG production

The sGAG production was evaluated in a DMMB (341088, Sigma, USA) assay, as previously described [29]. At the end of compression/H_2_O_2_ treatment, cells were reseeded in 6-well cell culture plates and incubated for 3 days. The culture medium of each sample was collected and 40 μl of the supernatant of each sample transferred to a 96-well microplate, after which 250 μl of DMMB solution was added. The DMMB-sGAG complex product was examined by an ELISA reader (Sunrise, TECAN, Switzerland) at the wavelength of 595 nm. The sGAG production activity of each sample was determined using a calibration curve of condroitin-6-sulfate (C4384, Sigma, USA). The sGAG production was normalized to cell numbers by a total DNA assay (sGAG to DNA ratio). Chondrocytes derived from more than three porcine stifles were used for the experiments. Five repeated measurements were performed for all samples.

### Alcian blue staining

After compression/H_2_O_2_ treatment, cells were reseeded into 4-well chamber-slides and cultured for 3 days. Chondrocytes were washed twice with PBS and fixed in 10% neutral buffered formalin (H121-08, Mallinckrodt Analytical, USA) for 30 min and then washed twice with PBS. Alcian blue (pH 1.0, Muto pure chemicals, Japan) was added for 30 min and cells were then washed in running water for 1 min. Nuclear fast red (1001210500, Merck, Germany) was added for 5 min and then washed in running water for 1 min. The cells were dehydrated in 2 changes of 95% alcohol and absolute alcohol (459844, Sigma, USA) for 1 min each. The sGAG content images were taken by using an IX71 inverted microscope equipped with a DP30BW digital camera system (Olympus, Japan). Chondrocytes derived from more than three porcine stifles were used for the experiments. Five repeated measurements were performed for all samples.

### Statistical analysis

The normality of variance of data was tested before statistical analysis. Statistically significant differences between the groups were determined by one-way ANOVA with Tukey’s post-hoc test. The results were expressed as mean ± standard deviation of the mean (SD) and considered significant when the *P*-value was <0.05. Statistical analysis was performed using the SigmaPlot version 12.3 software (Systat Software Inc., San Jose, USA).

## Results

### Aucubin shows no cytotoxic effects on chondrocytes

**Fig. 1:**
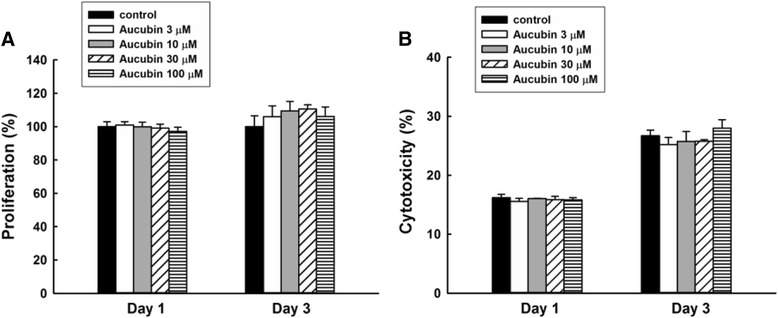
WST-1 and LDH tests of aucubin. Chondrocytes treated with 3, 10, 30 and 100 μM of aucubin for 1 and 3 days were examined with (**a**) WST-1 for cell proliferation and (**b**) LDH for aucubin cytotoxicity. Chondrocytes without aucubin treatment were set as the control group. Results shown represent mean ± SD obtained from five repeated measurements

As shown in Fig. 1, chondrocytes treated with 3, 10, 30, and 100 μM of aucubin showed no significant differences in cytotoxicity or cell proliferation activity compared to controls at 1 or 3 days, indicating that aucubin within the concentration range of 3–100 μM shows no significant cytotoxic effects on chondrocytes at either 1 or 3 days.

### Aucubin inhibits H_2_O_2_-induced ROS production in chondrocytes

**Fig. 2:**
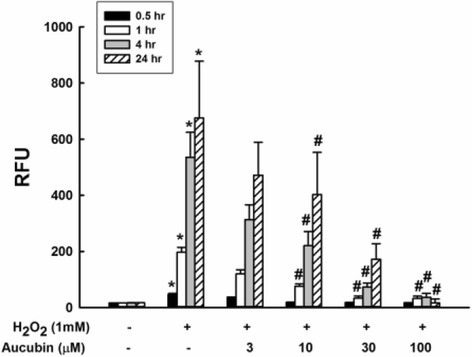
ROS scavenge test of aucubin. Chondrocytes pretreated with 0, 3, 10, 30 and 100 μM of aucubin for 0.5, 1, 4 and 24 h were stimulated with 1 mM H_2_O_2_ for 30 min. ROS production was measured by the intensity of the dye fluorescence and represented as relative fluorescence unit (RFU). Results shown represent mean ± SD obtained from five repeated measurements. *p < 0.05 compared with the control group. ^#^ *p* < 0.05 compared with the H_2_O_2_ group

**Fig. 3:**
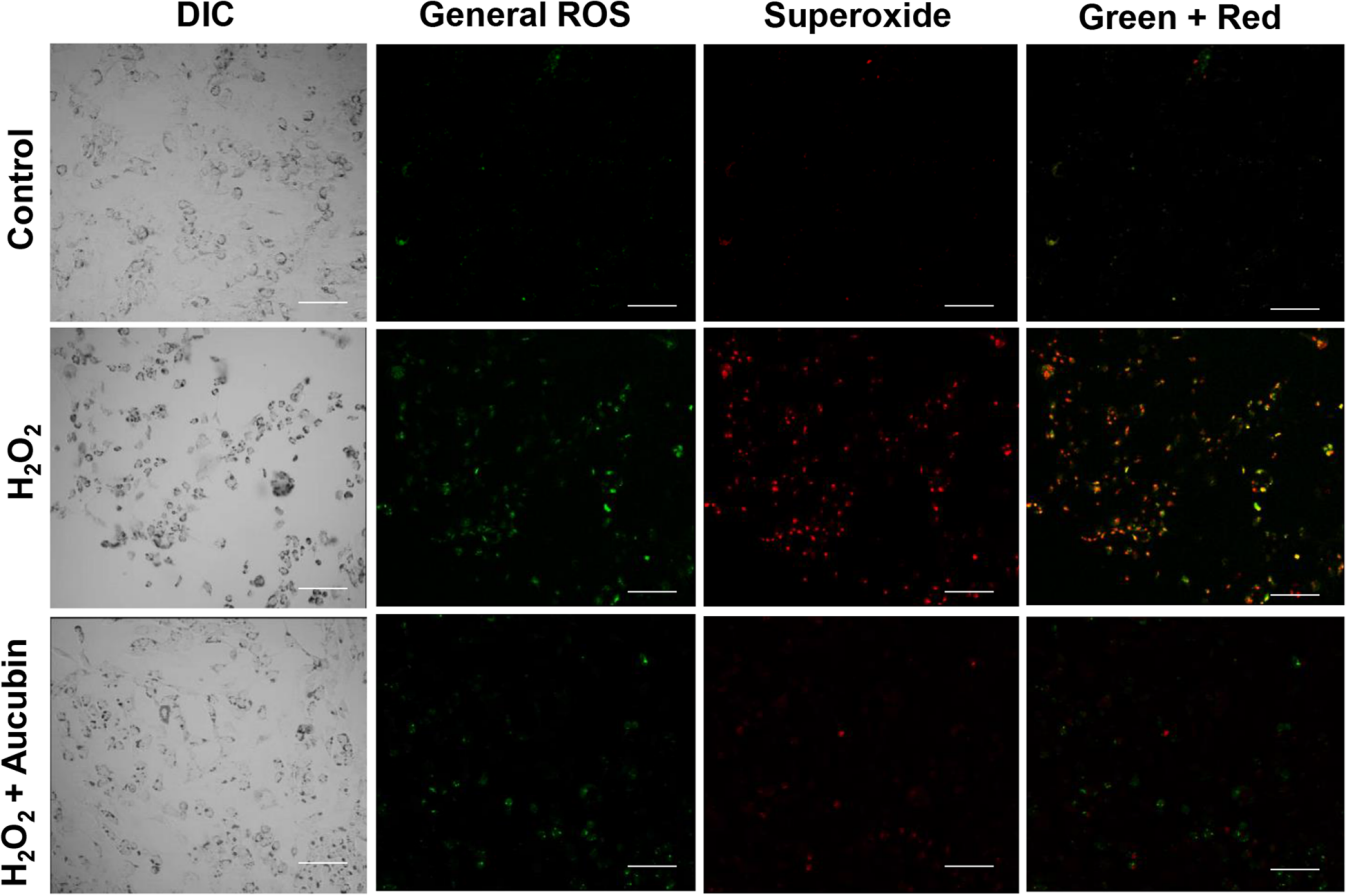
Fluorescence imaging of ROS production. Chondrocytes pretreated with 100 μM of aucubin for 24 h were then stimulated with 1 mM H_2_O_2_ for 30 min. The *green* and *red* colors are the fluorescence of general ROS (hydrogen peroxide, peroxynitrite, hydroxyl radicals, nitric oxide, peroxy radical) and superoxide, respectively. Chondrocytes without aucubin nor H_2_O_2_ treatment were set as the control group. *Scale bar* = 100 μm. (5 repeated measurements for each group)

As shown in Fig. 2, the ROS production was significantly increased in response to H_2_O_2_ stimulation. Chondrocytes treated with aucubin showed significant ROS scavenging effects after 1, 4 and 24 h of incubation with 10 to 100 μM aucubin in a dose-dependent manner. The ROS scavenge effect of aucubin was further confirmed through fluorescence imaging. As shown in Fig. 3, both the green and red fluorescence emissions, representing general ROS and superoxide induced by H_2_O_2_, respectively, were markedly reduced in fluorescence intensity and area after 24 h of 100 μM aucubin pretreatment. Since 100 μM aucubin showed the greatest scavenging effect with limited cytotoxicity among other aucubin concentrations, we used 100 μM aucubin in the subsequent experiments.

### Aucubin inhibits H_2_O_2_-induced caspase-3 activity on chondrocytes

**Fig. 4:**
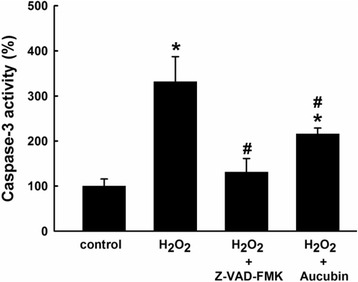
Effect of aucubin on caspase-3 activity. Chondrocytes pretreated with 100 μM of aucubin or Z-VAD-FMK as caspase inhibitor for 24 h were then stimulated with 1 mM H_2_O_2_ for 30 min. Chondrocytes without aucubin nor H_2_O_2_ treatment were set as the control group. Results shown represent mean ± SD obtained from five repeated measurements. *p < 0.05 compared with the control group. ^#^ *p* < 0.05 compared with the H_2_O_2_ group

After H_2_O_2_ treatment, the caspase-3 activity of chondrocytes showed a significant increase. However, chondrocytes treated with aucubin and caspase inhibitor Z-VAD-FMK markedly decreased the caspase-3 activity caused by H_2_O_2_ stimulation (Fig. 4).

### Aucubin inhibits H_2_O_2_-induced apoptosis and necrosis in chondrocytes

**Fig. 5:**
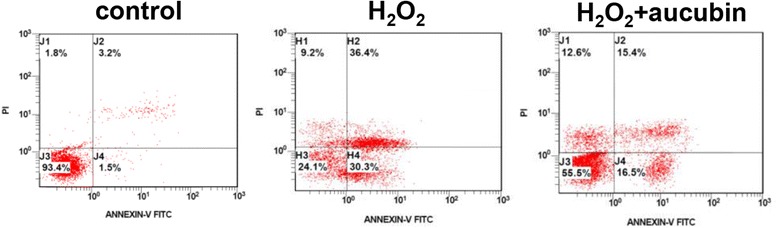
Annexin V/PI test of aucubin. Chondrocytes pretreated with 100 μM of aucubin for 24 h were then stimulated with 1 mM H_2_O_2_ for 30 min. Chondrocytes without aucubin nor H_2_O_2_ treatment were set as the control group. (5 repeated measurements for each group)

After H_2_O_2_ treatment, the percentage of apoptotic and necrotic chondrocytes were significantly increased compared to the control group. However, in the aucubin pretreated group, the percentages of chondrocytes in both early and late apoptosis states were significantly reduced compared to the H_2_O_2_ stimulated group (Fig. 5, Table 2).

**Table 2:** Quantitative results of Annexin V/PI test of aucubin

	Percentage of cell (%)			
	Normal	Early apoptosis	Late apoptosisi	Necrosis
Control	89.7 ± 9.5	2.5 ± 4.6	5.0 ± 3.8	3.1 ± 1.8
H_2_O_2_	25.5 ± 7.3^a^	29.4 ± 4.2^a^	32.2 ± 6.8^a^	14.3 ± 7.6^a^
H_2_O_2_ + aucubin	60.1 ± 10.4^a,b^	15.4 ± 5.3^a,b^	15.8 ± 4.2^a,b^	10.7 ± 5.3

Results shown represent mean ± SD obtained from 5 repeated measurements. ^a^ *p* <0.05 compared with the control group. ^b^ *p* < 0.05 compared with the H_2_O_2_ group

### Aucubin reverses H_2_O_2_-mediated and compression-mediated gene expression of ACAN and COL2A1 in chondrocytes

**Fig. 6:**
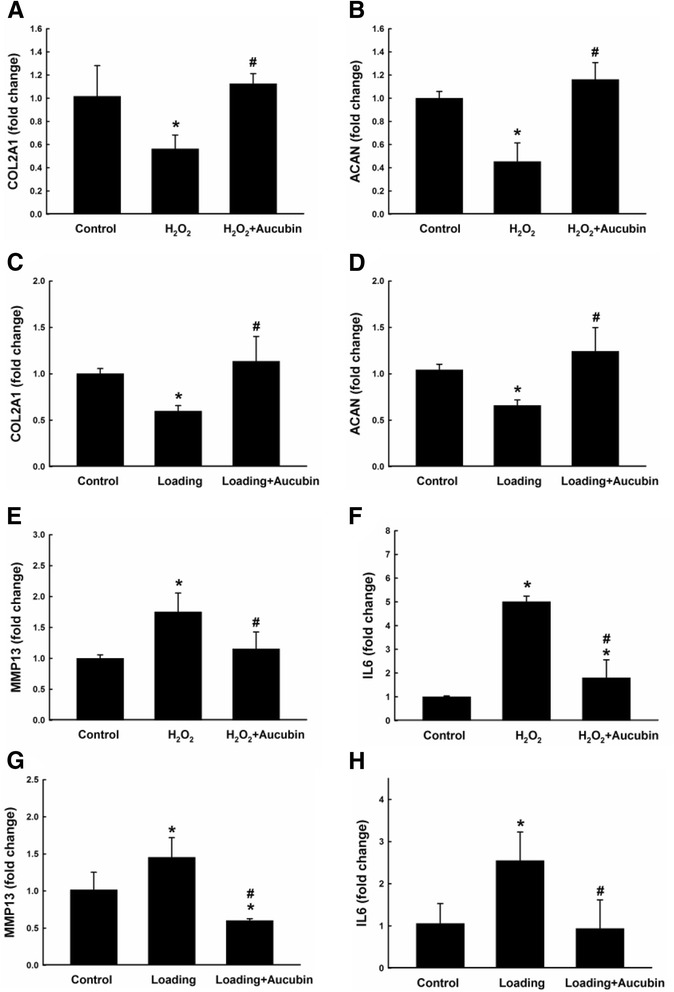
Gene expressions after stimulation. Gene expressions of COL2A1 (**a**, **c**), ACAN (**b**, **d**), MMP13 (**e**, **g**) and IL6 (**f**, **h**) were examined by RT-qPCR. Chondrocytes pretreated with 100 μM of aucubin for 24 h were then stimulated with 1 mM H_2_O_2_ (**a**, **b**, **e** and **f**) for 30 min, or 60 psi compression (**c**, **d**, **g** and **h**) for 24 h. Data from five independent biological replicates each with three technical replicates are expressed as fold change compared to untreated control and shown represent mean ± SD. Each target gene was normalized to GAPDH. *p < 0.05 compared with the control group. ^#^ *p* < 0.05 compared with the H_2_O_2_ or loading group

ACAN and COL2A1 are ECM related genes. As shown in the H_2_O_2_ model of Fig. 6a and b, the expression of both ACAN and COL2A1 was significantly down-regulated in the H_2_O_2_-treated group compared to the control group. However, pretreatment with aucubin reversed the expression of these genes back to the level of the control group and showed marked up-regulation compared to the H_2_O_2_-treated group. Similar results were shown in the compression model. The expression of both ACAN and COL2A1 was also markedly down-regulated in the loading group, though the aucubin pretreated chondrocytes had a significant up-regulation of both ECM component genes after compression stimulation compared to the control group (Fig. 6c and d).

### Aucubin reduces H_2_O_2_-mediated and compression-mediated gene expression of MMP13 and IL6 in chondrocytes

MMP13 is a catabolic gene in cartilage ECM homeostasis. As shown in Fig. 6e and g, in both H_2_O_2_ and compression models, the expression of MMP13 was up-regulated compared to the control group after stimulation. However, treatment with aucubin before H_2_O_2_ and compression stimulation maintained the expression of MMP13 at a comparable level to the control group. Similar results were shown in the expression of the proinflammatory gene IL6. As shown in Fig. 6f and h, IL6 was up-regulated compared to the control group in both models after stimulation. Moreover, the up-regulated IL6 expression was reversed to a similar level as the control group with the pretreatment of aucubin.

### Aucubin increases sGAG production in compression model but not after H_2_O_2_ stimulation

**Fig. 7:**
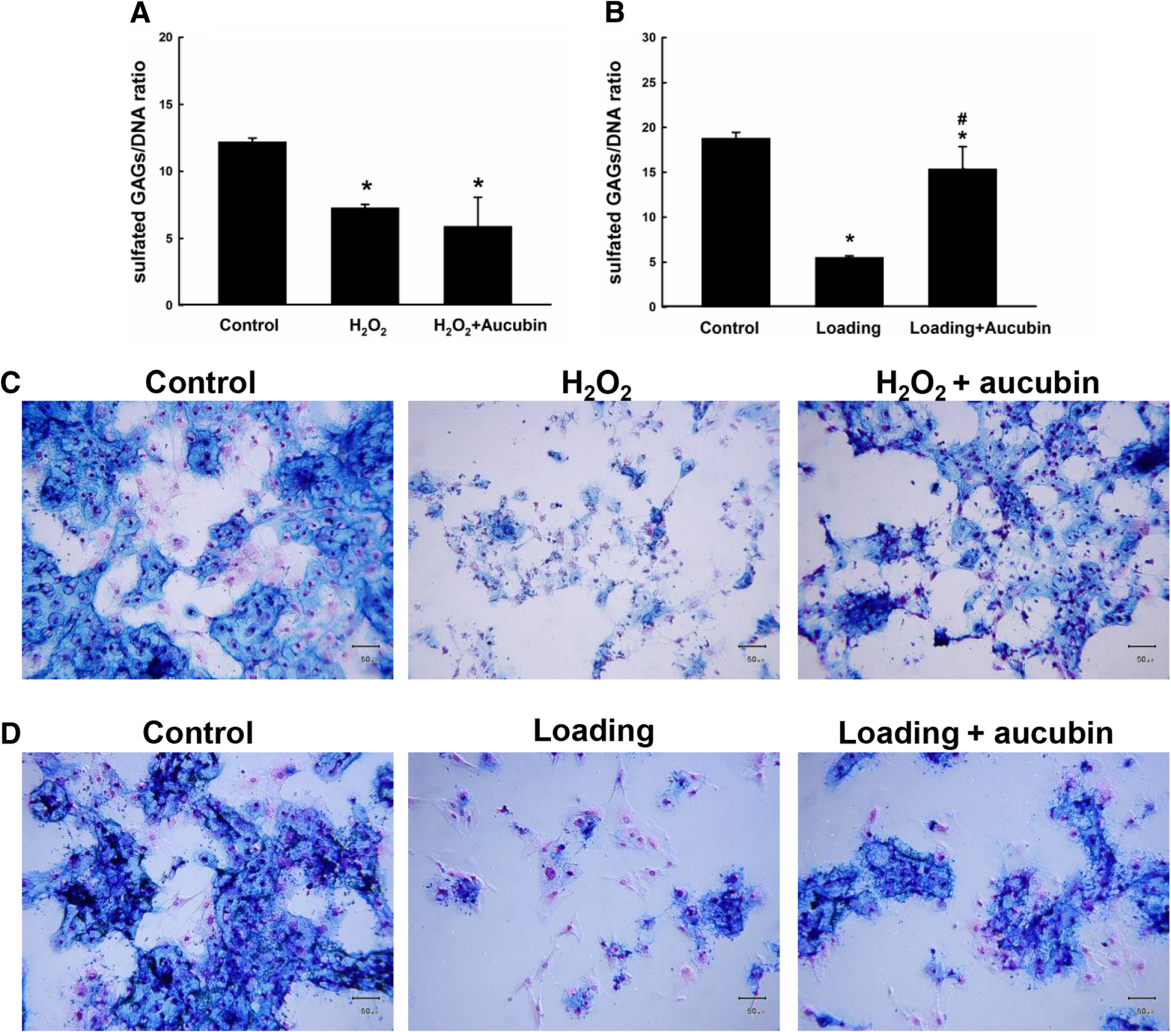
sGAG production and Alcian blue staining of GAG contents after stimulation. The ratio of sulfated-GAG to DNA of chondrocytes after stimulated with 1 mM H_2_O_2_ (**a**) for 30 min or compression (**b**) with 60 psi for 24 h. Alcian blue and nuclear fast red staining of the chondrocytes was performed after stimulated with (**c**) 1 mM H_2_O_2_ for 30 min or (**d**) compression with 60 psi for 24 h. *Scale bar* = 50 μm. Results shown represent mean ± SD obtained from five repeated measurements. *p < 0.05 compared with the control group. ^#^ *p* < 0.05 compared with the loading group

As shown in Fig. 7a, the sGAG to DNA ratio for the H_2_O_2_ treated group was significantly decreased compared to the control group. Treatment with aucubin showed no significant difference for the H_2_O_2_ treated group. However, the decreased sGAG to DNA ratio induced by compressive stress was significantly increased compared to the compression group (Fig. 7b).

### Aucubin increases the sGAG content after both H_2_O_2_ and compression stimulation

As shown in Fig. 7c and d, the alcian blue staining was positive in the control groups for both H_2_O_2_ and compression models. The H_2_O_2_ treated group, on the contrary, showed few areas of blue color with shrinking cell morphology. With aucubin pretreatment, some of the chondrocytes could maintain sGAG content. Similar results were also evident in the compression model.

## Discussion

Aucubin has significant antioxidant and radical scavenging properties in both in vivo and in vitro models [30], making it a potential therapeutic agent in oxidative stress-induced diseases. Aucubin has been shown to reduce ROS formation, malondialdehyde levels and β-galactosidase activity, and increase glutathione levels in UVB-irradiated human skin fibroblasts [31]. To evaluate the protective effects of aucubin in regards to OA, first we generated an osteoarthritic cell model with porcine chondrocytes by using 1 mM H_2_O_2_, which decreased 60% of chondrocytes proliferation activity but showed no significant or immediate cytotoxicity to untreated control (data not shown). In this study, 10–100 μM of aucubin caused no significant cytotoxicity or differences in cell proliferation activity after incubation with chondrocytes at 1 or 3 days (Fig. 1). After H_2_O_2_ stimulation, 10–100 μM of aucubin was a sufficient range to reduce ROS production dose-dependently after at least 1 h of pretreatment (Fig. 2). Pretreatment with 100 μM of aucubin also inhibited H_2_O_2_-induced caspase-3 activity (Fig. 4). Xue et al. reported that aucubin inhibited H_2_O_2_‐induced apoptosis in PC12 cells through regulation of the endogenous oxidant–antioxidant balance [27]. In this study, we confirmed the apoptosis inhibition properties of aucubin via Annexin-V/PI flow cytometry (Fig. 5). The percentage of apoptotic cells was significantly reduced when 100 μM of aucubin were used, which is consistent with the Xue et al. study and suggests that aucubin protects chondrocytes from cell apoptosis which is often observed during OA progression.

Aggrecan and type II collagen are the main components of chondrocyte ECM and provide cartilage with compressive and tensile resistance, respectively. In the OA cartilage, a loss of proteoglycans and decrease in type II collagen has been observed, leading to defective integrity of the cartilage [29]. In this study, both H_2_O_2_ and compressive loading caused significant down-regulations of ACAN and COL2A1, suggesting the stimulated chondrocytes were undergoing a degeneration process with weakened anabolic activity (Fig. 6). However, chondrocytes pretreated with aucubin showed a protective effect by reversing the H_2_O_2_/compression induced down-regulation of both ACAN and COL2A1 genes, which may implicate that aucubin has a beneficial effect on chondrocyte ECM production.

The expression of pro-inflammatory IL-6 inhibits the synthesis of aggrecan and is highly elevated in OA cartilage [32]. IL-6 expression directly contributes to the inhibitory effect of aggrecan through the Notch receptor [33], mediating induction of MMP-13 expression [34]. MMP-13 is a catabolic factor for cartilage ECM metabolism, degrading proteoglycans and type II collagen [35, 36]. Aucubin has been shown to inhibit TNF-alpha and IL-6 production in antigen-stimulated rat basophilic leukemia-2H3 mast cells [37]. Aucubin also has been demonstrated to reverse the increased gene and protein expression of MMP13, iNOS and COX-2 induced by IL-1β stimulation in rat chondrocytes [38]. In this study, aucubin significantly reversed the elevated gene expression of IL6 and MMP13 genes in the H_2_O_2_ and compression stimulated chondrocytes, consistently demonstrating the anti-inflammatory and anti-catabolic properties of aucubin in chondrocytes (Fig. 6).

About the single reference gene of GAPDH, in the previous study we had examined the gene expression of porcine chondrocytes and used GAPDH as reference gene for RT-qPCR [28]. After analysis the GAPDH gene showed a consistent Ct value among groups with different treatment. In the present study, we used the same method to isolate the porcine chondrocytes and observed the same phenomenon of GAPDH expressions in RT-qPCR results. The Ct values of GAPDH did not show a noticeable difference among chondrocytes stimulated with compression, H_2_O_2_, Aucubin or in control group. We suggest that treatments used in the present study did not affect the expression of GAPDH gene, thus, we used GAPDH as the house keeping gene in this study.

In the degenerated cartilage, a decrease in the content of chondroitin sulfate results in a decrease of water content that affects the capability of cartilage to absorb the external stress [39]. In this study, H_2_O_2_ and compression stimulation significantly reduced the sGAG production activity and decreased sGAG content. Chondrocytes pretreated with aucubin succeeded to maintain its sGAG content after both H_2_O_2_ and compression stimulation, and recover its sGAG production activity after compression stimulation. However, aucubin failed to maintain the sGAG production activity after H_2_O_2_ stimulation (Fig. 7). According to these data, we suggest that the protective effects of aucubin may be overwhelmed by the oxidative stress of H_2_O_2_. The oxidative stress may lead to decrease the sGAG production activity of chondrocytes. Nevertheless, aucubin still has beneficial ability to maintain sGAG content of chondrocytes after H_2_O_2_ and compression stimulation.

## Conclusions

This study demonstrated that aucubin can reduces ROS production, caspase-3 activity, and cell apoptosis. Aucubin can protect porcine chondrocytes from H_2_O_2_ and compression-induced dysregulation of COL2A1, ACAN, IL6 and MMP13 gene expressions. Additionally, aucubin helps porcine chondrocytes maintain sGAG contents after H_2_O_2_ and compressive stimulation. These results indicated that aucubin has protective effects in an osteoarthritic chondrocyte model induced by H_2_O_2_ and mechanical stimulus.

## Abbreviations

ADAMTS: A disintegrin and metalloproteinase with thrombospondin motifs
DMEM: Dulbecco’s modified eagle’s medium
ECM: Extracellular matrix
GAPDH: Glyceraldehyde-3-phosphate dehydrogenase
IL-6: Interlukin-6
LDH: Lactate dehydrogenase
MMP13: Matrix metalloproteinase 13
OA: Osteoarthritis
PBS: Phosphate buffered saline
ROS: Reactive oxygen species
RT-qPCR: Reverse transcription quantitative polymerase chain reaction
sGAG: Sulfated-glycosaminoglycan
